# Traumatic Brain Injury in Qatar: Age Matters—Insights from a 4-Year Observational Study

**DOI:** 10.1155/2013/354920

**Published:** 2013-07-22

**Authors:** Moamena El-Matbouly, Ayman El-Menyar, Hassan Al-Thani, Mazin Tuma, Hany El-Hennawy, Husham AbdulRahman, Ashok Parchani, Ruben Peralta, Mohammad Asim, Ahmed El-Faramawy, Ahmad Zarour, Rifat Latifi

**Affiliations:** ^1^Weill Cornell Medical College, Doha 24144, Qatar; ^2^Clinical Research, Trauma Surgery Section, Hamad General Hospital, Doha 3050, Qatar; ^3^Clinical Medicine, Weill Cornell Medical College, Doha 24144, Qatar; ^4^Trauma Surgery Section, Hamad General Hospital, Doha 3050, Qatar; ^5^Department of Surgery, Arizona University, P.O. Box 245005, Tucson, AZ, USA

## Abstract

*Background*. Overall traumatic brain injury (TBI) incidence and related death rates vary across different age groups. *Objectives*. To evaluate the incidence, causes, and outcome of TBI in adolescents and young adult population in Qatar. *Method*. This was a retrospective review of all TBIs admitted to the trauma center between January 2008 and December 2011. Demographics, mechanism of injury, morbidity, and mortality were analyzed in different age groups. *Results*. A total of 1665 patients with TBI were admitted; the majority were males (92%) with a mean age of 28 ± 16 years. The common mechanism of injury was motor vehicle crashes and falls from height (51% and 35%, resp.). TBI was incidentally higher in young adults (34%) and middle age group (21%). The most frequent injuries were contusion (40%), subarachnoid (25%), subdural (24%), and epidural hemorrhage (18%). The mortality rate was 11% among TBI patients. Mortality rates were 8% and 12% among adolescents and young adults, respectively. The highest mortality rate was observed in elderly patients (35%). Head AIS, ISS, and age were independent predictors for mortality. *Conclusion*. Adolescents and adults sustain significant portions of TBI, whereas mortality is much higher in the older group. Public awareness and injury prevention campaigns should target young population.

## 1. Introduction

Traumatic brain injuries (TBIs) are one of the main public health problems, leading to a high morbidity and mortality rate in the United States [1]. The latest report of the Center for Disease Control (CDC) shows that TBIs pose a huge burden on health system resulting in 1.7 million cases annually with 52,000 deaths, over 250,000 hospitalizations annually and over a million emergency department visits [2]. TBI-related deaths are responsible for approximately one third of all injury-related deaths in the USA [3], with disproportionately high fatalities among males compared to females [2]. Moreover, long-term TBI-related disabilities result in reduced quality of life. Current estimates suggest that approximately 3.2–5.3 million persons live with a long-term disability due to brain injury [4]. Recent studies showed that the cost of TBIs, only in the United States, is estimated to be between $48.3 and 76.3 billion annually [5, 6]. Most common causes of TBI are falls (35.2%), motor vehicle crashes (MVCs) (17.3%), struck by/against events (16.5%), and assaults (10%) [2]. In the developing countries, the incidence of motor-bikes-related TBIs are significantly increasing as compared to other causes [7]. TBI is also a leading cause of death and disability in war zones [8]. Falls are the frequently observed cause of TBI among young children (2–4 yrs), whereas, MVC and falls are almost equally responsible for TBI in older children [9]. Previous report from Qatar showed a substantial increase in the incidence of head injuries across all ages [10]. Herein, we aimed to evaluate the causes and trend of TBI in our populations.

## 2. Methods

Data were collected retrospectively for all traumatic brain injuries patients admitted to the Level I trauma Center at Hamad General Hospital in Qatar between January 2008 and December 2011. Demographics, mechanism of injuries, associated injuries, operative procedures, morbidity, and mortality were analyzed. Glasgow coma scale (GCS) at scene, Injury Severity Score (ISS), and head Abbreviated Injury Score (AIS) were also documented. Patients were categorized into 8 groups with a 10-year interval apart. This study was approved by the medical research center at HMC, Qatar, and IRB no. 12175/12.

### 2.1. Statistical Analysis

Data were presented as proportions, mean ± standard deviation (SD), or median as appropriate. Baseline demographic characteristics, clinical presentation, and outcomes were compared according to age groups using the one-way ANOVA test for continuous variables and Pearson chi-square (*Χ* ^2^) test for categorical variables. Multivariate logistic regression analysis was performed to determine the predictors for mortality among TBI patients. A significant difference was considered when the 2-tailed *P* value was less than 0.05. Data analysis was carried out using the Statistical Package for Social Sciences version 18 (SPSS Inc., USA).

## 3. Results 

**Figure 1:**
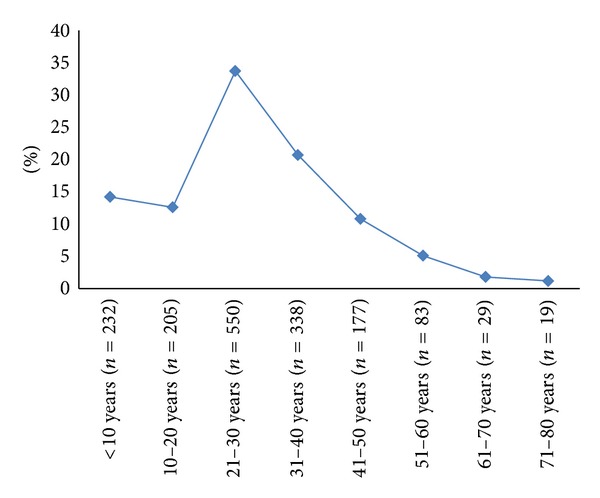
Head injury in different age groups.

From January 2008 to December 2011, a total of 1665 TBIs patients were included in the study. The Majority of patients were males (92%) and were expatriates (80%) with mean age of 28  ±  16 years. We excluded 72 (4%) patients with incomplete data. TBI was incidentally higher among adults (21–30 yrs, 34%) and middle age group (31–40, 21%). Teenagers (10–20 yrs) represented about 13% of the TBI patients (Figure 1). The most common mechanism of head injury was MVC (51%), followed by falls from height (35%) (Table 1).

The majority of patients were intubated in the emergency department (50%) and at the scene (36%). Tracheostomy was performed in 5% of patients, open reduction and internal fixation (ORIF) were needed in 12%, and craniotomy was needed in 11% cases (Table 1). Craniotomy was frequently performed in patients aged 51–60 years (16%) and 41–50 years (16%). Craniectomies were most common in 31–40 age group (33%) as well as 41–50 age group (23%) patients. The most frequent brain injuries were brain contusion (40%), subarachnoid hemorrhage (25%), subdural hemorrhage (24%), and epidural hemorrhage (18%) (Table 2). Fracture of the vault and base of the skull were observed in 37.5% and 37% of cases, respectively (Table 2). Moreover, injuries of lung (19%), lower (10%) and upper (11%) extremities, and rib fracture (13%) were the most common associated injuries (Table 2). Only few patients developed complications such as pneumonia (4%), ARDS (0.5%), and sepsis (0.1%). 

Table 3 demonstrates the main mechanisms of head injury by different age groups. MVC was the most common mechanism of injury among individuals of young ages. Fall was the second common MOI in these age groups. Further, TBI due to all-terrain vehicles (ATV) accidents was frequently observed in teenagers (11%) followed by 41–50 yrs (7.5%), 31–40 yrs (7%), and 21–30 yrs (5%) age groups, respectively.

According to severity of injury, 414 (25%) patients had severe TBI (GCS ≤ 8), 97 (5.8%) had moderate (GCS 9–12), and 1091 (65.5%) had TBIs (GCS ≥ 13), respectively. Among teenagers (10–20 yrs) and young adults (21–30 yrs), lower mean GCS was observed at scene (Table 4). Whereas the mean head AIS (3.5 ± 0.9) was the highest among elderly population (71–80 yrs) (Table 4). Median period of mechanical ventilation was 6.5 (1–16) days in the 61–70 age group and 4 (1–24) days in the adolescent age group. Median hospital length of stay [11 (1–66)] was the highest among elderly population.

**Figure 2:**
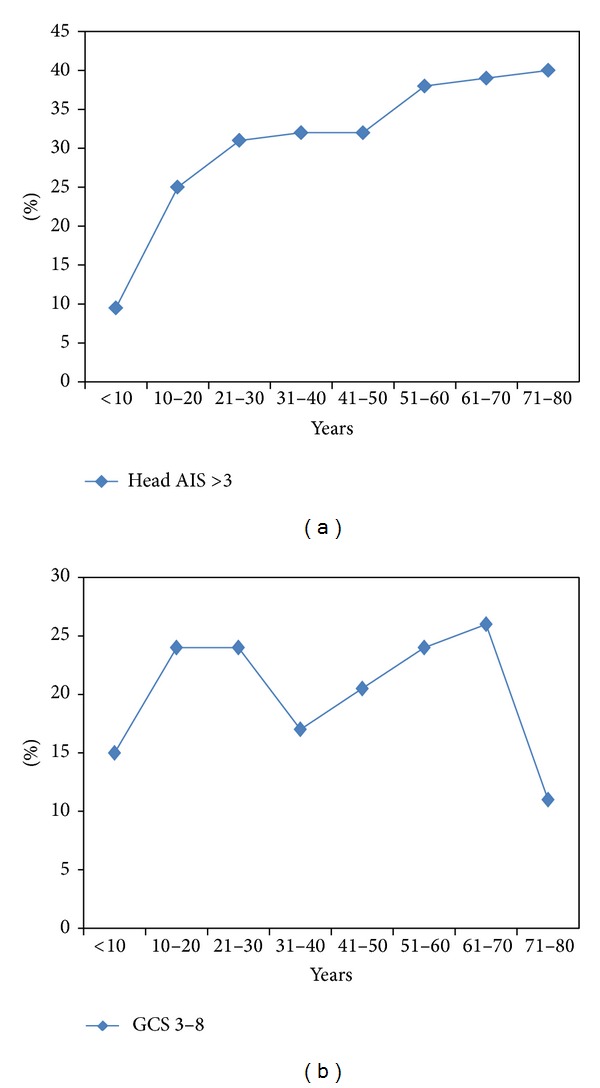
Traumatic brain injury in different age groups based on head abbreviated injury severity (AIS) and Glasgow Coma Scale (GCS).

Figure 2 shows an increasing trend of severe head injury (head AIS > 3) with age. Low GCS (8-3) had 2 peaks, one in age between 10 and 30 and another one at age between 60 and 70 years.

**Figure 3:**
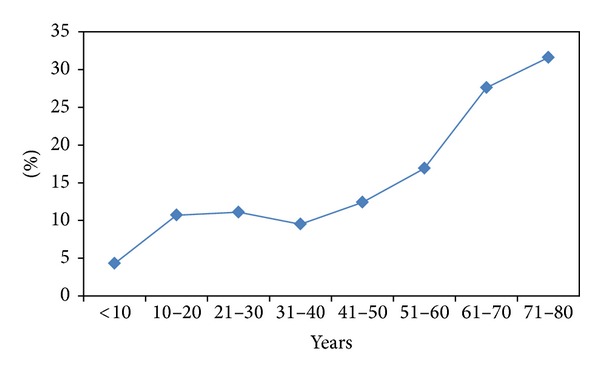
Mortality among TBI patients according to age (*P* = 0.001).

The overall mortality was 11% among TBI patients. An increasing trend of mortality was observed with respect to age (Figure 3). The highest mortality rate (35%) was observed in elderly (71–80 yrs) patients followed by 61–70 age group with 21% mortality. Mortality rate was comparable among teenagers (8.1%) and young adults (11.7%), respectively.  Table 5 demonstrates multivariate logistic regression analysis to determine the predictors for mortality among TBI patients. Head AIS, ISS, age, and GCS were the independent predictors for mortality in TBI patients.

## 4. Discussion

Traumatic brain injury (TBI) is defined as damage to the brain resulting from external mechanical force, such as rapid acceleration or deceleration, impact, blast waves, or penetration [11]. TBI is a leading cause of death and disability around the globe [12]. It represents a major social, economic, and public health problem worldwide [11]. Although, in developed countries, the incidence of fall-related TBI is increasing with progressive age, thus the median age of people with head injuries has increased [11], and there is a high incidence of TBIs among young population [13]. The most vulnerable age groups for TBI are children of five to nine years and elderly population [14]. TBI is the leading cause of brain damage in children and young adults [15]. Around 85% of all traumatic injuries in children are associated with TBI, either alone or with other injuries [16]. TBI is the highest in young adults aged 15 to 24 years and is higher in men than women in all age groups [17]. Majority of our patients (92%) were males. Our findings revealed that TBI outcomes vary greatly with age. The highest incidence of TBI was in 20–29 years age group (followed by 30–39 years and less than 10 years old age group. Men suffer twice as many TBIs as women do and have a fourfold risk of fatal head injury [14], and males account for two-thirds of childhood and adolescent head trauma [18]. Socioeconomic status also appears to affect TBI rates; people with lower levels of education and employment and lower socioeconomic status are at greater risk [15]. It is generally agreed that a TBI with a GCS of 13 or above is mild, 9–12 is moderate, and 8 or below is severe [19–21]. The mean GCS at scene, the mean ISS, and head AIS were comparable among adolescent and young adult populations. In our study, diffuse axonal injury was found in only 2% of patients, while brain edema was presented in 10% of patients. In our study brain contusions were presented in 40% of the patients. Diagnosis of TBI is based on lesion circumstances, clinical evidence, neurological examination, and radiologic examination. The preferred radiologic test in the emergency setting is computed tomography (CT) scan. CT scan evaluation was done in all of our patients. According to CDC, one-third of all injury deaths involved TBI [22]. Overall injury and TBI-related death rates vary across different age groups. Our study showed that head AIS, ISS, and age were the main independent predictors for mortality in TBI patients. 

Highest injury and TBI-related mortality rates were observed in adults aged 20–24 years (76.9 per 100,000) and among individuals aged ≥75 years (173.2 per 100,000) [22]. In the USA, the fatality rate was estimated to be 21% by 30 days after TBI [23]. A study on Iraq war soldiers found that severe TBI carries a mortality of 30–50% [8]. However, mortality rate has declined in developed nations due to improved treatments and trauma management systems [24]. The fraction of those who died after being hospitalized with TBI fell from almost half in the 1970s to about a quarter at the beginning of the 21st century [25]. This decline in mortality has led to a concomitant increase in the number of individuals living with TBI-related disabilities [26]. In our study, the mortality is increasing with age to reach a peak of 35% in elderly population. However the fraction of severe traumatic brain injury defined by GCS < 8 was similar among both groups. Our data also shows that 51% of TBI are caused by MVC, 35% due to falls, 6% by motor cycles/ATV, and 5% from fall of heavy objects. Prevention of MVC or the improvement of medical care can reduce both the incidence and severity of TBI. The severity of injuries in a vehicle crash could be minimized through compliance of safety measures such as use of seat belts, child safety seats [27] and motorcycle helmets, [28], and presence of roll bars and airbags [29].

There are number of limitations of this study. One of the limitations is the retrospective nature of the study. We did not go into details about the pathophysiology of injury and evolution of injury cascades. This could be useful in managing preventative measures to decrease the incidence of traumatic head injuries. Our patients represent Qatar population for moderate and severe TBIs, as we are the only tertiary center with full EMS, trauma, and neurosurgery services. However, we cannot extrapolate these results to mild TBIs, as many of those treated in other state hospitals are discharged from the emergency department or even not shown at all. Also no functional outcome data is available from this retrospective review.

## 5. Conclusion

TBI is a common injury in Qatar. About half of the victims are adolescents and young adults. A quarter of this age group is severely injured, and the mortality among them represents one-fifth of overall mortality. Half of the injuries were MVC-related. Therefore, a multifaceted injury prevention program is urgently warranted.

## Figures and Tables

**Table 1:** Demographics, injury mechanism, and interventions in TBI patients.

Total number	*n* = 1655 (%)
Male	1546 (92)
Age (mean ± SD)	28 ± 16
Nationality	
Qatari	333 (20)
Non-Qatari	1304 (80)
Main mechanisms of injury	
Blunt injuries	1654 (99)
MVCs	847 (51)
Fall from height	590 (35)
Motor cycles and other vehicles	98 (6)
Fall of heavy objects	82 (5)
Others	37 (3)
Interventions	
ETT Intubation	566 (34)
Emergency department	279 (49.8)
On-scene	200 (35.7)
Referring hospital	48 (8.6)
CT head	1655 (100)
Tracheostomy	81 (5)
ORIF	203 (12)
Craniotomy	178 (11)

MVC: motor vehicle rashes; ORIF: open reduction and internal fixation; ARDS: acute respiratory distress syndrome.

**Table 2:** Traumatic brain injuries and associated injuries.

	*n* (%)
Head injuries	
Subdural hemorrhage	402 (24.3)
Subarachnoid hemorrhage	407 (24.6)
Extradural hemorrhage	298 (18)
Extra-axial hemorrhage	51 (3)
Intraventricular hemorrhage	40 (2)
Intracerebral hemorrhage	34 (2)
Intrahemispheric hemorrhage	4 (0.2)
Pneumocephalus	148 (9)
Brain edema	160 (10)
Fracture to the vault	624 (37.5)
Fracture to the base	621 (37)
Brain contusion	666 (40)
Diffuse axonal injury	35 (2)
Associated injuries	
Liver	88 (5)
Lung	315 (19)
Spleen	96 (6)
Bowel	22 (1)
Kidney	41 (2.5)
Cardiac	5 (0.3)
Diaphragmatic	4 (0.2)
Aortic	9 (0.5)
Rib fracture	220 (13)
Pelvic fracture	90 (5)
Lower extremities injuries	168 (10)
Upper extremities injuries	185 (11)
Complications	
Pneumonia	65 (4)
ARDS	9 (0.5)
Sepsis	2 (0.1)
Mortality	182 (11)

**Table 3:** Mechanism of injury by age groups.

	<10	10–20	21–30	31–40	41–50	51–60	61–70	71–80
MVC	48 (20.7%)	144 (70.2%)	313 (57.2%)	162 (48.2%)	85 (48.9%)	48 (58.5%)	17 (60.7%)	8 (42.1%)
Fall from height	156 (67.2%)	28 (13.7%)	167 (30.5%)	126 (37.5%)	59 (33.9%)	26 (31.7%)	10 (35.7%)	11 (57.9%)
Fall of heavy objects	16 (6.9%)	6 (2.9%)	24 (4.4%)	16 (4.8%)	13 (7.5%)	6 (7.3%)	1 (3.6%)	0 (0%)
ATV	8 (3.4%)	23 (11.2%)	28 (5.1%)	24 (7.1%)	13 (7.5%)	1 (1.2%)	0 (0%)	0 (0%)
Others	4 (1.7%)	4 (2.0%)	15 (2.7%)	8 (2.4%)	4 (2.3%)	1 (1.2%)	0 (0%)	0 (0%)

**Table 4:** Characteristics of patients of different age groups based on the injury severity, length of hospital stay and ventilator days.

Age groups	GCS (on-scene)^†,∗^	ISS^†,∗∗^	Head AIS^†,∗∗^	Length of stay^††,∗∗^	Ventilation days^††,∗∗^
<10	13.2 ± 3.7	10.3 ± 7.8	2.8 ± 0.7	2 (1–83)	4 (1–64)
10–20	11.8 ± 4.4	18.2 ± 9.1	3.3 ± 0.8	9 (1–193)	4 (1–24)
21–30	11.8 ± 4.5	18.7 ± 10.6	3.3 ± 0.8	8 (1–380)	3 (1–29)
31–40	12.7 ± 3.9	18.3 ± 9.6	3.3 ± 0.8	7.5 (1–380)	4 (1–56)
41–50	12.5 ± 4.1	18.03 ± 9.2	3.3 ± 0.9	8 (1–410)	3 (1–36)
51–60	12.3 ± 4.2	19.7 ± 10.5	3.4 ± 1.0	8.5 (1–159)	3 (1–17)
61–70	12.2 ± 4.2	18.2 ± 9.6	3.4 ± 0.9	7 (1–47)	6.5 (1–16)
71–80	12.8 ± 4.0	17.8 ± 10.2	3.5 ± 0.9	11 (1–66)	2 (1–8)

GCS: Glasgow Coma Scale; ISS: injury severity score; head AIS: abbreviated injury score. *P value 0.07, **P value < 0.001, ^†^mean ± SD; ^††^median (range).

**Table 5:** Predictors for in-hospital mortality in traumatic head injury.

	Odd ratio	95% confidence interval	*P* value
Age	1.01	1.001–1.036	0.036
Head AIS	2.45	1.33–4.54	0.004
GCS	0.17	0.11–0.28	0.001
ISS	1.13	1.09–1.17	0.001
Lung injury	0.68	0.39–1.22	0.20
Liver injury	0.71	0.30–1.71	0.45
Craniotomy	0.85	0.44–1.66	0.65
